# International disparities in conservation priorities are more complicated than Global North–Global South divisions

**DOI:** 10.1098/rsbl.2024.0571

**Published:** 2025-03-19

**Authors:** Yolanda Mutinhima, Lovemore Sibanda, Betty J. Rono, Salum Kulunge, David Kimaili, Amy J. Dickman, Emily Madsen, Lessah Mandoloma, Jessica Tacey, Shorna Allred, Darragh Hare

**Affiliations:** ^1^ Department of Wildlife Ecology and Conservation, Chinhoyi University of Technology, Chinhoyi, Zimbabwe; ^2^ Centre for Sustainability Transitions, Stellenbosch University, Stellenbosch, South Africa; ^3^ Department of Biology, Oxford University, Oxford, UK; ^4^ Wildlife Conservation Research Unit, The Recanati-Kaplan Centre, Department of Biology, Oxford University, Tubney, UK; ^5^ Cheetah Conservation Project Zimbabwe, Dete, Zimbabwe; ^6^ Department of Zoology and Entomology, Rhodes University, Grahamstown, South Africa; ^7^ Department of Wildlife Management, Sokoine University of Agriculture, Morogoro, Tanzania; ^8^ Tanzania Wildlife Management Authority, Morogoro, Tanzania; ^9^ Department of Sociology and Anthropology, South Eastern Kenya University, Kitui, Kenya; ^10^ Department of Geography and Environment, University of North Carolina at Chapel Hill, Chapel Hill, NC, USA; ^11^ Department of Natural Resources and the Environment, Cornell University, Ithaca, NY, USA

**Keywords:** 30 × 30, conservation conflict, global biodiversity framework, inclusive conservation, politics

## Abstract

Two enduring ideological divisions in biodiversity conservation concern whether conservation should prioritize (i) the interests of people or wild animals and (ii) the interests of individual animals or groups of animals. Public debates suggest that people living in the Global North more strongly prioritize the interests of wild animals over people and the interests of individual animals over groups of animals. To examine this possibility, we measured and compared conservation priorities across 10 international publics living in rural and urban areas of sub-Saharan Africa, the United States of America (USA) and the United Kingdom (UK). Overall, distant respondents (i.e. living in the UK, USA and urban sub-Saharan Africa) more strongly prioritized the interests of wild animals over people and the interests of individual animals over groups of animals. Moreover, variation among local publics (i.e. living in high-biodiversity areas of rural sub-Saharan Africa) was greater than among distant publics. Our findings illuminate how ideological divisions may complicate international biodiversity conservation, especially around controversial topics such as culling, hunting, transloaction and protected-areas management. Policies and programmes more acceptable to distant people may be less acceptable to local people, creating difficulties for decision-makers charged with balancing biodiversity conservation alongside the values, needs, interests and concerns of multiple publics.

## 1. Introduction

Reversing or halting global biodiversity loss will require monumental effort [1]. While some biodiversity declines could be addressed locally, collective international efforts will be essential for identifying priority areas for global conservation, conserving transboundary ecosystems and populations, reducing drivers of biodiversity loss that originate in distant consumption and coordinating the flow of financial and technical resources [2–6]. However, as with any collective endeavour, ideological divisions between and among conservation scientists and practitioners can impede efforts to conserve biodiversity [7–9]. Two of these enduring ideological divisions concern what biodiversity conservation should prioritize. The first concerns whether biodiversity conservation should prioritize the interests of people or the interests of wild animals [10–12]. The second concerns whether biodiversity conservation should prioritize the interests of individual wild animals or groups of wild animals [13,14].

Regarding the first division, some scholars argue for a transition towards a system of conservation that centres on the rights and needs of people living in biodiversity-rich areas, sometimes referred to as inclusive conservation [15–17]. While this approach has the potential to reduce injustices associated with traditional area-based models of conservation [18,19], some argue that conservation’s primary focus must remain centred on biodiversity and its intrinsic value rather than its relationship with and utility to people [20–23]. There is, therefore, a risk that actions taken to conserve biodiversity could exacerbate historic injustices and undermine the values needs, interests and concerns of people living in biodiversity-rich areas [11,17].

The second division reflects long-standing debates over whether conservation should prioritize the interests of individual wild animals or groups of wild animals [24,25]. Proponents of ‘compassionate conservation’ argue that conventional approaches that focus on groups of wild animals (e.g. populations, species or communities comprising multiple species) overlook ethical obligations to prevent the suffering of individual animals [25–27]. Further, some proponents argue that it is morally unacceptable to override the interests of an individual wild animal for the benefit of a larger group of wild animals [27]. Others argue that prioritizing outcomes for individual wild animals over groups of animals or ecological wholes would lead to ineffective conservation actions, and disregards cultural diversity in human–wildlife relationships, especially for societies with deep-rooted cultural traditions of harvesting wild animals [28–32].

Recent international policy discourses in print and social media raise the possibility of a geographical dimension to these divisions. For example, there may be differences in how people living in the Global North think about conservation priorities compared with rural people living in the Global South. One high-profile example came when Mokgweetsi Masisi, then President of Botswana, vividly posited such divisions, claiming that Europeans value the lives of wild animals over the lives of African people [33]. Although President Masisi’s statement implied a clash of fundamental values based on geography (i.e. Europe versus Africa), surprisingly little data exist to evaluate this possibility. Understanding the extent to which different publics agree or disagree on conservation priorities could help inform decisions on controversial issues in biodiversity conservation [7,34].

Here we present the results of a questionnaire study measuring whether members of 10 international publics (four from the Global North and six from the Global South) would prioritize (i) the interests of people or the interests of wild animals and (ii) the interests of individual animals or groups of animals when their interests clash. Our objective was to test whether there are differences in conservation priorities across ecologically and socioculturally diverse locations.

## 2. Methods

**Figure 1.**
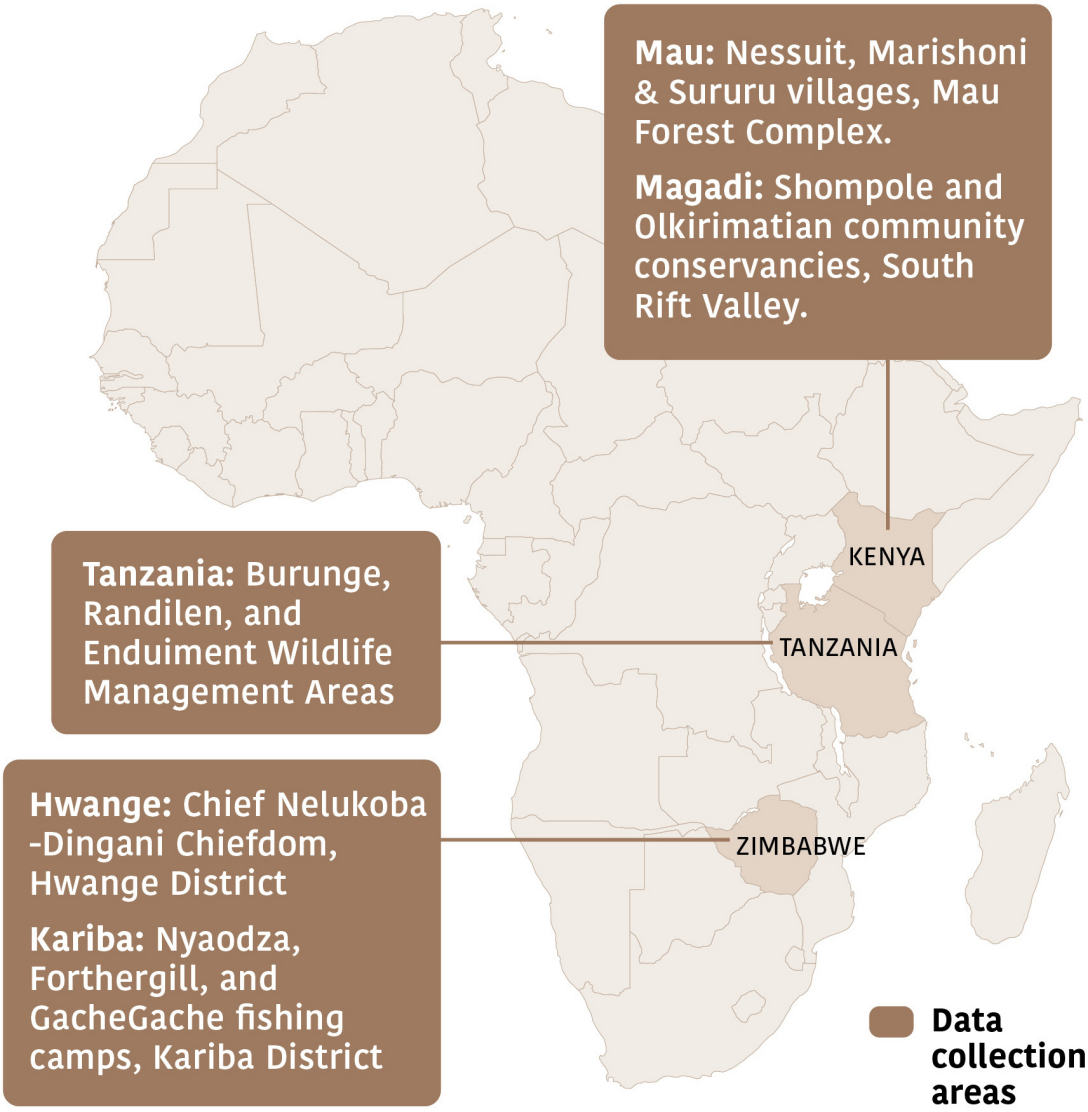
Locations in rural Kenya (Mau and Magadi), Tanzania, and Zimbabwe (Hwange and Kariba), where we collected in-person data. We collected data from the UK, USA, and urban areas of sub-Saharan Africa online.

Between October 2022 and September 2023, we measured what members of 10 publics think about conservation priorities in sub-Saharan Africa, the United States of America (USA) and the United Kingdom (UK). We used in-person questionnaires to collect data from people living in five rural areas of sub-Saharan Africa: Magadi (southern Kenya), Mau (southern Kenya), Hwange (north-western Zimbabwe), Kariba (northern Zimbabwe) and Burunge, Enduiment, and Randilen Wildlife Management Areas (WMAs), northern Tanzania (figure 1). We used online questionnaires to collect data from people living in urban areas of sub-Saharan Africa, rural and urban areas of the UK and rural and urban areas of the USA. We, therefore, have data from five ‘local’ publics (i.e. people who live in high-biodiversity areas of rural sub-Saharan Africa) and five ‘distant’ publics (i.e. people who live outside of high-biodiversity areas of rural sub-Saharan Africa).

We asked respondents to answer two questions corresponding to enduring ideological divisions over conservation priorities: whether they would prioritize the interests of people or the interests of wild animals (hereafter, people–animals) and whether they would prioritize the interests of individual animals over the interests of groups of animals (hereafter, individuals–groups). For people–animals, we asked: *if the interests of wild animals clash with the interests of people, which do you think should be prioritized?* Respondents indicated their response using a five-point Likert-type scale from strongly prioritize people to strongly prioritize wild animals, with a neutral value of prioritize neither wild animals nor people and an additional option of I do not know. For individuals–groups, we asked: *if the interests of individual animals clash with the interests of groups of animals, which do you think should be prioritized?* Respondents indicated their responses using a five-point Likert-type scale from strongly prioritize individual animals to strongly prioritize groups of animals, with a neutral value of prioritize neither individual animals nor groups of animals and an additional option of I do not know. We also recorded respondents’ age, gender and level of formal education, variables that consistently predict values and attitudes towards conservation [35]. To minimize priming effects, we randomized the order in which respondents answered all questions. In line with best practices for community involvement in research [36], we held in-person meetings between October and December 2023 with community members in all five rural African study sites to help understand and interpret findings. Perspectives of community members are reflected in the discussion. More detailed methods, including sample descriptions, are in the electronic supplementary material.

### (a) Data analysis

We collected data from 3834 respondents across all 10 locations. We removed all *I do not know* responses to leave only responses on each scale, and responses from people who reported non-binary gender identities because the numbers were too small to include as a factor in our models. For people–animals, we removed 246 *I do not know* responses and 17 gender non-binary responses, and for individuals–groups we removed 444 *I do not know* responses and 17 gender non-binary responses. This left 3571 people–animals responses for analysis (median per location = 358, minimum = 129 and maximum = 562; table 1) and 3373 individuals–groups responses (median per location = 326, minimum = 109 and maximum = 534; table 2).

**Table 1.** Description of sample for differences in whether respondents would prioritize the interests of people or the interests of wild animals. SSA: sub-Saharan Africa, USA: the United States of America, UK: the United Kingdom

characteristic	USA urban	USA rural	UK urban	UK rural	urban SSA	Tanzania	Mau	Magadi	Kariba	Hwange
sample size (*n* = 3571)	294	129	312	135	497	521	286	403	432	562
***age***										
18−30	69(23.5%)	30(23.3%)	66(21.2%)	30(22.2%)	134 (26.9%)	178(34.2%)	92(32.2%)	87(21.6%)	173(40%)	125 (22.2%)
31−40	82(27.9%)	18(13.9%)	75(24%)	25(18.5%)	133(26.8%)	138(26.5%)	75(26.2%)	132(32.8%)	121(28%)	114(20.3%)
41−50	41(13.9%)	19(14.7%)	58(18.6%)	20(14.8%)	82(16.5%)	115(22.1%)	68(23.8%)	95(23.6%)	89(20.6%)	100(17.8%)
51−60	27(9.2%)	11(8.5%)	67(21.5%)	26(19.3%)	86(17.3%)	62(11.9%)	28(9.8%)	52(12.9%)	30(6.9%)	107(19.1%)
Over 60	75(25.5%)	51(39.5%)	46(14.7%)	34(25.2%)	62(12.5%)	28 (5.4%)	23(8%)	37(9.2%)	19(4.4%)	116(20.6%)
***gender***										
female	143(48.6%)	73(56.6%)	146(46.8%)	82(60.7%)	243(48.9%)	271(52%)	123(43%)	224(55.6%)	228(52.8%)	340(60.5%)
male	151(51.4%)	56(43.4%)	166(53.2%)	53(39.3%)	254(51.1%)	250(48%)	163(57%)	179(44.4%)	204(47.2%)	222(39.5%)
***formal education***										
no formal education	6(2%)	1(0.8%)	4(1.3%)	1(0.7%)	1(0.2%)	112(21.5%)	56(19.6%)	305(75.7%)	19(4.4%)	48(8.5%)
primary school	19(6.5%)	12(9.3%)	3(0.9%)	1(0.7%)	3(0.6%)	284(54.5%)	75(26.2%)	50(12.4%)	97(22.5%)	176(31.3%)
secondary school	91(31%)	52(40.3%)	105(33.7%)	54(40%)	122(24.5%)	103(19.8%)	109(38.1%)	39(9.7%)	315(72.9%)	320(56.9)
college or university	133(45.2)	56(43.4%)	163(52.2%)	60(44.4%)	314(63.2%)	19(3.6%)	45(15.7%)	9(2.2%)	1(0.2%)	17(3%)
postgraduate (e.g. master’s, PhD)	45(15.3%)	8(6.25)	37(11.9%)	19(14.1%)	57(11.5%)	3(0.6%)	1(0.4%)	0(0%)	0(0%)	1(0.3%)

**Table 2.** Description of sample for differences in whether respondents would prioritize the interests of individual wild animals or the interests of groups of wild animals. SSA: sub-Saharan Africa, USA: the United States of America, UK: the United Kingdom

characteristic	USA urban	USA rural	UK urban	UK rural	urban SSA	Tanzania	Mau	Magadi	Kariba	Hwange
sample size (*n* = 3373)	272	109	289	123	472	508	285	363	418	534
***age***										
18−30	62(22.8%)	27(24.8%)	65(22.5%)	31(25.2%)	130(27.5%)	173(34.0%)	91(31.9%)	77(21.2%)	165(39.5%)	124(23.2%)
31−40	81(29.8%)	16(14.7%)	69(23.9%)	22(17.9%)	126(26.7%)	133(26.2%)	79(27.7%)	121(33.3%)	120(28.7%)	108(20.2%)
41−50	39(14.3%)	16(14.7%)	55(19%)	17(13.8%)	78(16.5%)	113(22.2%)	62(21.7%)	89(24.5%)	85(20.3%)	95(17.8%)
51−60	25(9.2%)	8(7.3%)	61(21.1%)	23(18.7%)	78(16.5%)	62(12.2%)	28(9.8%)	47(13%)	30(7.2%)	98(18.4%)
over 60	65(23.9%)	42(38.5%)	39(13.5%)	30 (24.4%)	60(12.7%%)	27(5.3%)	25(8.8%)	29(8%)	18(4.3%)	109(20.4%)
***gender***										
female	125(46%)	60(55%)	135(46.7%)	75(61%)	228(48.3%)	262(51.6%)	123(43.2%)	197(54.3%)	219(52.4%)	324(60.7%)
male	147(54%)	49(45%)	154(53.3%)	48(39%)	244(51.7%)	246(48.4%)	162(56.8%)	166(45.7%)	199(47.6%)	210(39.3%)
***formal education***										
no formal education	6(2.2%)	2(1.8%)	2(0.7%)	1(0.8%)	1(0.2%)	107(21.06%)	55(19.3%)	275(75.8%)	18(4.3%)	47(8.8%)
primary school	17(6.3%)	9(8.3%)	4(1.4%)	1(0.8%)	3(0.6%)	278(54.7%)	75(26.3%)	44(12.1%)	92(22%)	166(31.1%)
secondary school	83(30.5%)	39(35.8%)	94(32.5%)	52(42.3%)	114(24.2%)	100(19.7%)	109(38.2%)	36(9.9%)	307(73.4)	305(57.1%)
college or university	122(44.9%)	52(47.7%)	154(53.3%)	51(41.5%)	298(63.1%)	20(3.9%)	45(15.8%)	8(2.2%)	1(0.3%)	15(2.8%)
postgraduate (e.g. master’s, PhD)	44(16.1%)	7(6.4%)	35(12.1%)	18(14.6%)	56(11.9%)	3(0.6%)	1(0.4%)	0	0	1(0.2%

We used ordinal regression to quantify associations between respondents’ location and their answers to the questions people–animals and individuals–groups. We fitted two separate models, one with people–animals as the response variable and one with individuals–groups as the response variable. For each model, we included respondents’ place of residence as the predictor of interest, respondents’ age, gender and highest level of formal education as control variables [35,37], and a random effect to account for multiple respondents from the same households. We used likelihood ratio tests (LRTs) to identify significant predictors (*p* < 0.05) for each model, and Tukey tests adjusted for unequal sample sizes to test for differences between levels of significant predictors with more than two levels. We report full details of all statistical results in the electronic supplementary material.

We analysed our data in R v. 4.2.3 [38], using the ‘ordinal*’* package [39] to fit models, ‘RVAideMemoire’ for LRTs, ‘emmeans’ [40] to calculate marginal predictions and conduct Tukey tests, ‘Likert’ [41] to visualize raw data and ‘ggplot2’ [42] to graph model predictions and 95% CIs [43]. Data and code are available from Figshare [44].

## 3. Results

### (a) People–animals

**Figure 2.**
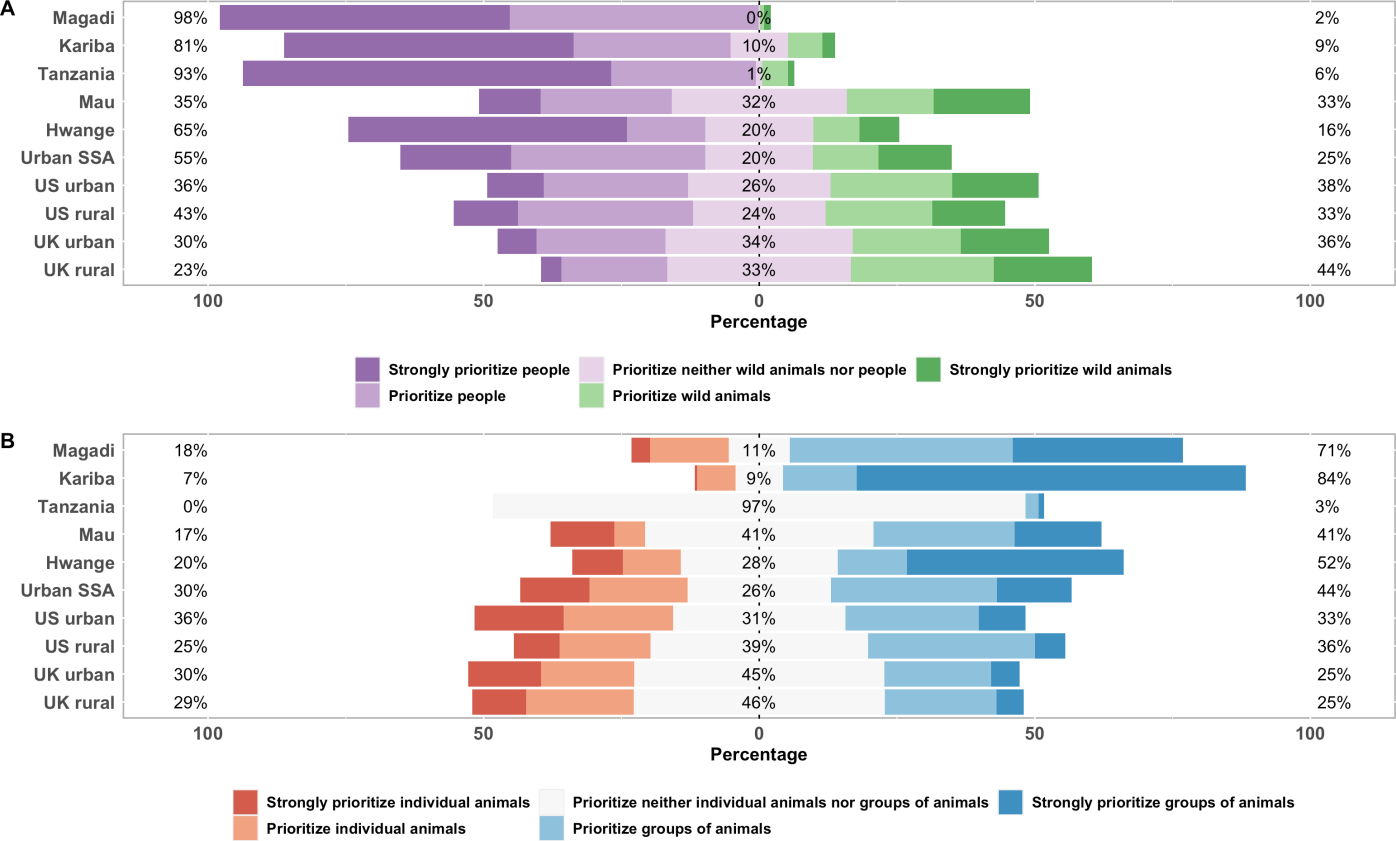
Conservation priorities among 10 international publics. Bars are grouped by respondents from each location, and colours show distribution of responses. Panel A displays how respondents would prioritize the interests of people or the interests of wild animals, and percentages show combined proportions of respondents from each location who indicated that they would strongly prioritize or prioritize people (left), prioritize neither wild animals nor people (middle) or prioritize or strongly prioritize wild animals, (right), after excluding ‘I do not know’ responses. Panel B displays how respondents would prioritize the interests of individual animals or the interests of groups of animals, and percentages show combined proportions of respondents from each location who indicated that they would strongly prioritize or prioritize individual animals (left), prioritize neither individual animals nor groups of animals (middle) or prioritize or strongly prioritize groups of animals (right), after excluding ‘I do not know' responses. ‘Urban SSA’ denotes respondents from urban areas of sub-Saharan Africa.

**Figure 3.**
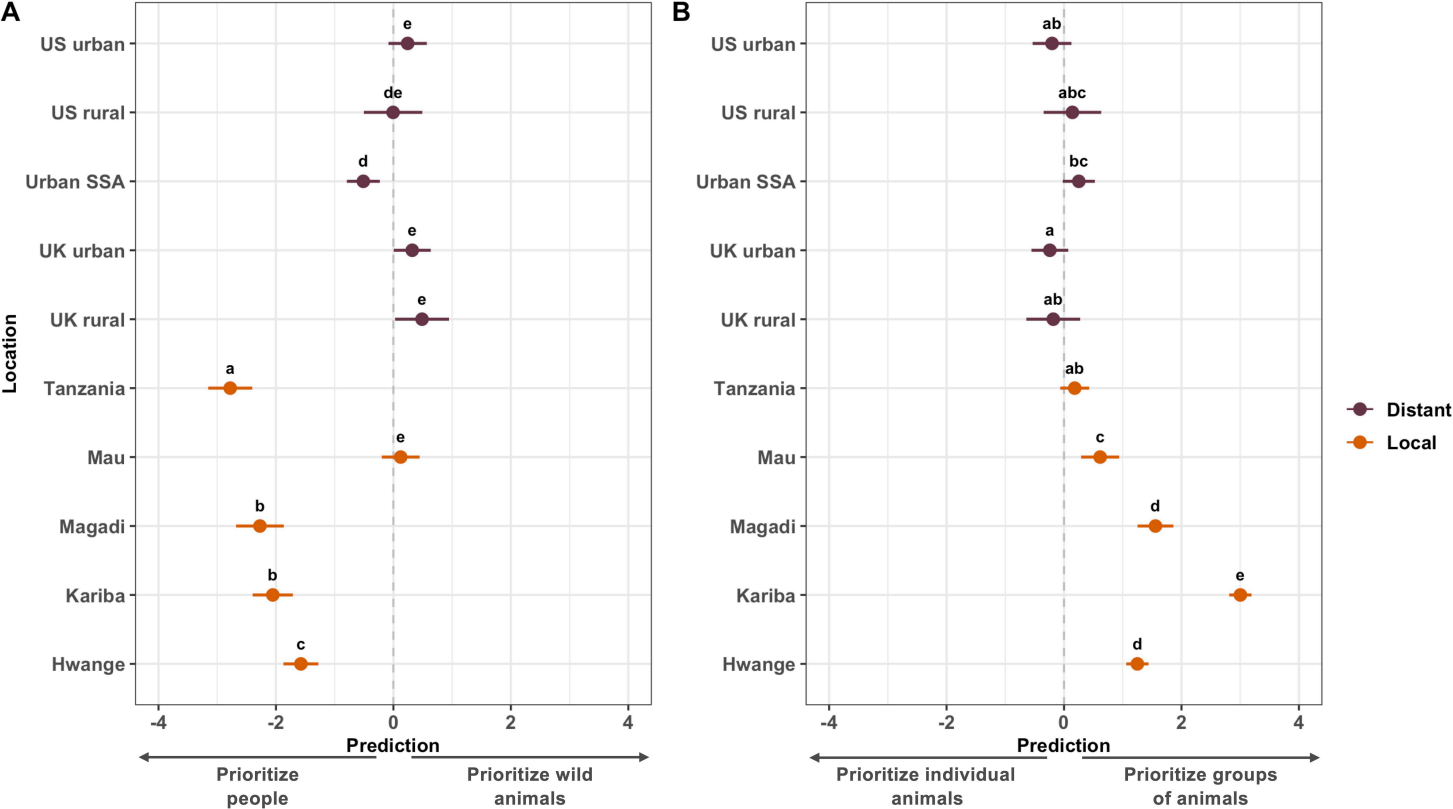
Conservation priorities vary across 10 international publics. Points show model-derived estimates for how respondents from 10 locations would prioritize (A) the interests of people or the interests of wild animals and (B) the interests of individual animals or groups of animals. Error bars show 95% CIs, and colours show whether locations are local (i.e. in high biodiversity areas of rural sub-Saharan Africa) or distant (i.e. outside of high biodiversity areas of rural sub-Saharan Africa). Within panels, points sharing letters are not statistically significantly different. ‘Urban SSA’ denotes respondents from urban areas of sub-Saharan Africa.

We found a statistically significant association between respondents’ place of residence and whether they would prioritize the interests of people or wild animals (LRT = 692.17, d.f. = 9, *p* < 0.001; electronic supplementary material). Prioritizing the interests of wild animals over the interests of people was strongest among respondents from rural areas of the UK, followed by urban areas of the UK, urban areas of the USA, Mau, rural areas of the USA, urban areas of sub-Saharan Africa, Hwange, Kariba, Magadi and Tanzania (figures 2A and 3A). The largest difference was between respondents from rural areas of the UK and respondents from Tanzania (Tukey test, difference [s.e.] = 3.27 [0.22], *p* < 0.001; electronic supplementary material).

### (b) Individuals–groups

We found a statistically significant association between respondents’ place of residence and whether they would prioritize the interests of individual animals or groups of animals (LRT = 650.01, d.f. = 9, *p* < 0.001; electronic supplementary material). Prioritizing the interests of groups of animals over the interests of individual animals was strongest among respondents from Kariba, followed by Magadi, Hwange, Mau, urban areas of sub-Saharan Africa, Tanzania, rural areas of the USA, rural areas of the UK, urban areas of the USA and urban areas of the UK (figures 2B and 3B). The largest difference was between respondents from Kariba and those from urban areas of the UK (Tukey test, difference [s.e.] = 3.26 [0.13], *p* < 0.001; electronic supplementary material).

## 4. Discussion

We investigated what members of 10 international publics think about whether conservation should prioritize people or wild animals and individuals or groups of animals. Our results reveal differences in conservation priorities that can be partially, but not completely, characterized as Global North–Global South divisions. Instead, differences in both priorities can be characterized as between local publics (i.e. living in biodiversity-rich areas in rural sub-Saharan Africa) and distant publics (i.e. living outside of biodiversity-rich areas in rural sub-Saharan Africa). However, we also found substantial diversity in priorities among areas of rural sub-Saharan Africa that are focal points for international efforts to conserve biodiversity.

Our results partially substantiate the notion that people in the Global North prioritize the interests of wild animals over the interests of people more strongly than do people in the Global South, for example, as suggested in the former President of Botswana’s public statement [33]. This finding brings into sharp focus a potentially important ideological division relevant to current international debates as tensions emerge around the social justice implications of area-based conservation targets under the Convention on Biological Diversity’s post-2020 Global Biodiversity Framework [8]. Proposals to continue traditional approaches that focus on protecting areas with the highest biodiversity can amplify the historical injustices associated with area-based conservation, which have marginalized people living in high-biodiversity areas [17,24,45,46]. When people living in high-biodiversity areas perceive that conservation policies and programmes prioritize the interests of biodiversity—particularly charismatic or potentially dangerous wild animals—above their own needs, they can reject conservation efforts and, in some cases, retaliate in ways that ultimately undermine conservation objectives [47].

However, people–animals priorities among people from urban areas in sub-Saharan Africa were, overall, more similar to responses from the UK and the USA than to respondents from all but one rural African location (figure 3A). This suggests that differences in this priority may be more accurately characterized as local–distant than Global North–Global South. The rural African exception was Mau, where responses were not significantly different from rural or urban areas of the UK or the USA (figure 3A). This finding is particularly surprising given ongoing tensions between the Ogiek people and the Kenyan Wildlife Service over restricted access to the Mau Forest, which could be seen as giving greater priority to biodiversity than people [48,49].

A plausible explanation for local–distant differences in the people–animals priority is that local respondents may have more first-hand experience of how people’s interests can conflict with those of wild animals, particularly over shared and increasingly limited resources such as land and water [50–52]. Competition between people and wild animals in high-biodiversity areas of Africa can result in injuries or even fatalities among people living next to wildlife as well as severe damage to livelihoods [52,53]. Compared with local respondents, distant respondents may be less familiar with scenarios where the interests of people and wild animals fundamentally clash and, therefore, less fully appreciate risks associated with living alongside dangerous wild animals or the extent and stakes of competition for limited space and resources in rural sub-Saharan Africa [52,54,55].

Prioritizing individual animals over groups of animals was stronger among respondents from the UK, USA and urban sub-Saharan Africa than among respondents from most other locations (figure 3B). One possible explanation is that animal protection and animal rights movements have more traction in distant locations [56]. Tensions between the interests of individual wild animals and populations of wild animals are evident in highly visible media coverage of contemporary polarized debates over hunting and culling in Africa. Examples include opposition from organized advocacy groups to importing rhino-hunting trophies into the USA [57], despite evidence that hunting can have positive effects on rhino populations [58]; proposals in Namibia and Zimbabwe to cull populations of large herbivores such as elephants amid escalating conflicts over scarce resources in periods of prolonged drought [59,60]; and legal hunting in Tanzania of elephants that move between Tanzania and Southern Kenya (where hunting is illegal; [61,62]). Zimbabwe’s controversial proposed elephant cull would take place partially in Hwange National Park, next to where our Hwange respondents live. Some elephant hunts in the Kenya–Tanzanian borderlands that provoked fierce debate took place in Enduiment Wildlife Management Area, where some of our Tanzanian respondents live (figure 1).

The conservation rationales for both trophy hunting and culling assume that benefits to people matter and that removing an individual wild animal can benefit populations of wild animals [59]. Our findings suggest that these types of reasoning generally resonate more strongly with people living in rural African locations than people living further away. Moreover, our findings could help explain why opposition to trophy hunting from the Global North is not matched in some African locations where hunting occurs [34,63]. In more distant locations, public discourse often focuses on whether or not it is ethically acceptable to kill a wild animal for sport or to benefit groups of animals [24,64]. During community meetings in rural areas of Tanzania and Zimbabwe, discussions on the acceptability of hunting focused less on the ethics of hunting individual animals and more on what (and how much) direct benefit local people could receive from trophy hunting, for example, through employment, meat, revenue and attenuating human–wildlife conflict by regulating populations of dangerous wild animals.

As with people–animals, differences in individuals–groups priorities may be more accurately characterized as local–distant rather than Global North–Global South. The strength of this priority among respondents from urban areas of sub-Saharan Africa was not significantly different from urban or rural areas of the USA or rural areas of the UK (figure 3B). However, a simple local–distant division could not explain why responses from Tanzania were not significantly different from rural areas of the UK, rural or urban areas of the USA or rural areas of sub-Saharan Africa (figure 3B). Ninety-seven per cent of respondents from Tanzania selected the neutral option (prioritize neither individual animals nor groups of animals, figure 2B) and none selected either prioritize or strongly prioritize individual animals. This unusual distribution, with most responses in the middle of the scale and no responses in the left half of the scale, could help explain similarities in model predictions between Tanzania and locations where responses were more widely distributed (figure 2B). During community meetings, we learned that the predominance of neutral responses among Tanzanian respondents might reflect local beliefs that all animals are entitled to exist, so prioritizing individuals or groups is equivalent to interfering with God’s work.

Although people–animals priorities in Mau and individuals–groups priorities in Tanzania align closely with priorities in some distant locations (figure 3A), the specific underlying reasons may be different. For example, for distant respondents from the UK and USA, more strongly prioritizing individual animals over groups of animals may be the influence of anthropomorphism and pet culture, which cast animals as companions with human-like personalities. Moreover, celebrity animals in media coverage and conservation campaigns in the Global North draw attention to specific individuals, presenting a narrative that conservation is about preserving individual animal lives rather than populations, communities, species or ecosystems [65–67]. However, the Ogiek people (the predominant tribe in our Mau sample) consider wildlife to be an integral part of their human existence and have cultural beliefs that assign intrinsic value even to wild animals that are frequently involved in negative interactions with people. Aggregate responses to closed-ended questionnaire items can reveal similarities in what people living in very different ecological and social circumstances think about conservation priorities (figure 3) but cannot capture potentially very profound cultural differences underpinning those responses.

While our results generally reveal local–distant divisions, we also found substantial differences in people–animals and individuals–groups priorities among rural African (i.e. local) locations. Indeed, for both priorities, we found much larger variation among local publics than among distant publics (figure 3). These local–local differences exist even within countries. For example, although Mau and Magadi are only approximately 300 km apart by road, we found differences in the distribution of responses between these locations (figure 2) as well as statistically significant differences in both priorities (figure 3). These differences may potentially stem from cultural differences, with respondents from Mau primarily Ogiek and Magadi primarily Maasai. However, respondents from Tanzania were also primarily Maasai, and both conservation priorities were significantly different between Magadi and Tanzania (figure 3), indicating that differences between rural African locations cannot be explained by broad cultural identity alone.

Overall, our findings provide new insights into how members of 10 international publics would prioritize the interests of people or the interests of wild animals and the interests of individual animals or groups of animals when their interests clash. We believe our results open an avenue for further inquiry to provide a more detailed view of what people across societies think about these complicated issues. For example, future research could employ more granular methods than our single-item measures, and evaluate how people evaluate trade-offs when presented with specific scenarios describing people–animals or individual–groups clashes, such as culling, hunting, translocations or protected areas management. Differences in survey modality (online and in person) may have introduced response biases that we could not account for. Moreover, ‘groups of animals’ is open to several possible interpretations not defined in our questionnaire item, and it is possible that individual–groups priorities might differ depending on how many animals are in the group, and whether the group comprises a single species or multiple species. Future research could also test for associations between socio-demographic factors and conservation priorities. While we found no significant associations between respondents’ gender, age or level of formal education, the two conservation priorities that we measured (electronic supplementary material), other sociodemographic factors such as economic status, exposure to conservation policies, cultural values concerning wildlife and trust in institutions may matter [68–70]. We encourage researchers to explore additional points of divergence and convergence between distant and local publics in terms of ongoing controversies in international conservation that might be underpinned by ideological differences, for example, hunting, culling, translocations, wildlife trade, wild meat consumption and area-based conservation.

Our findings shed new light on urgent debates in international biodiversity conservation by revealing that enduring ideological differences between and among conservation scientists and practitioners are reflected in what people think about conservation priorities across 10 international publics. Specifically, global efforts to conserve biodiversity may be complicated because policies and programmes that are more acceptable to distant people may be less acceptable to local people, and *vice versa*, increasing conflicts between local and distant groups and creating difficulties for decision-makers charged with balancing biodiversity conservation alongside the values, needs, interests and concerns of multiple publics [45]. By better understanding ideological similarities and differences, research can inform difficult decisions on thorny topics and help anticipate, attenuate or avoid impediments to international efforts to conserve biodiversity. By openly recognizing ideological and geographical divisions, the international conservation community can discuss how to reconcile divergent positions to achieve more effective, inclusive international conservation efforts.

## Data Availability

A preprint of the article as well as the data and R-scripts used in this study are available at [44]. Supplementary material available online [71].
